# RAAWC-UNet: an apple leaf and disease segmentation method based on residual attention and atrous spatial pyramid pooling improved UNet with weight compression loss

**DOI:** 10.3389/fpls.2024.1305358

**Published:** 2024-03-11

**Authors:** Jianlong Wang, Junhao Jia, Yake Zhang, Haotian Wang, Shisong Zhu

**Affiliations:** ^1^ School of Computer Science and Technology, Henan Polytechnic University, Jiaozuo, China; ^2^ School of Computer and Information Engineering, Henan Normal University, Xinxiang, China

**Keywords:** apple leaf and disease, CBAM, Resnet, ASPP, weight compress

## Abstract

**Introduction:**

Early detection of leaf diseases is necessary to control the spread of plant diseases, and one of the important steps is the segmentation of leaf and disease images. The uneven light and leaf overlap in complex situations make segmentation of leaves and diseases quite difficult. Moreover, the significant differences in ratios of leaf and disease pixels results in a challenge in identifying diseases.

**Methods:**

To solve the above issues, the residual attention mechanism combined with atrous spatial pyramid pooling and weight compression loss of UNet is proposed, which is named RAAWC-UNet. Firstly, weights compression loss is a method that introduces a modulation factor in front of the cross-entropy loss, aiming at solving the problem of the imbalance between foreground and background pixels. Secondly, the residual network and the convolutional block attention module are combined to form Res_CBAM. It can accurately localize pixels at the edge of the disease and alleviate the vanishing of gradient and semantic information from downsampling. Finally, in the last layer of downsampling, the atrous spatial pyramid pooling is used instead of two convolutions to solve the problem of insufficient spatial context information.

**Results:**

The experimental results show that the proposed RAAWC-UNet increases the intersection over union in leaf and disease segmentation by 1.91% and 5.61%, and the pixel accuracy of disease by 4.65% compared with UNet.

**Discussion:**

The effectiveness of the proposed method was further verified by the better results in comparison with deep learning methods with similar network architectures.

## 1 Introduction

The apple leaf is an important organ for the growth and development of apples. Apple is also the most grown fruit in northern China because of its high nutritional value, containing high levels of calcium, iron, zinc and other trace elements (Su et al., 2022). According to Shenzhen Daochuang Intelligence, China’s annual apple bagging output will reach 36.8 million tons in 2023. A key factor affecting apple yield is leaf photosynthetic area. However, its cultivation often breeds different diseases. Apples grow many diseases during cultivation and leaf diseases are a common plant disease (van Bruggen et al., 2016). The diseases are caused by fungi, bacteria, or viruses and can affect leaf respiration, which in turn affects apple growth and yield (Lee et al., 2020). Therefore, the quick detection of apple leaf diseases and precise spraying of pesticides according to the size of the leaf disease area are essential to guarantee the healthy growth of apples (Sun et al., 2021). Precision disease control techniques also play a decisive role in securing apple yields (Tang et al., 2023).

Traditional apple leaf and disease detection methods usually rely on manual visual identification or capture of pests to determine the likelihood of disease occurrence (Liu and Wang, 2021). The method involves high labor costs, lengthy time consumption, and is easily influenced by subjective factors (Li et al., 2021). With the development of image segmentation technology, traditional segmentation includes the Canny Edge Detection Algorithm (Xizhen et al., 2021), the Region Growing Algorithm (Jin et al., 2018), the Watershed Algorithm (Jin et al., 2018), and so on. These methods primarily concentrate on the local pixel relationships and can easily lead to the misconception of local optimization. Traditional segmentation techniques tend to produce discontinuous or incomplete segmentation results when applied to images with complex textures or shapes (Lu et al., 2023b). Moreover, it requires manual adjustment of certain parameters, which can be challenging for non-professionals. Lychee picking robots using artificial intelligence algorithms to proactively remove obstacles have been proposed, which provide an intelligent technology that reduces labor costs (Wang et al., 2023).

The combination of metaheuristics and machine learning methods is also an important research direction. The enhanced version of the firefly algorithm (FA) makes a great contribution to the prevention of overfitting in network training (Bacanin et al., 2021). A hierarchical feature selection method based on genetic algorithm for handwritten word recognition is proposed, which uses a hierarchical feature selection model to optimize the handwritten word images and extract the local and global features (Malakar et al., 2020). The genetically guided best artificial flora algorithm is proposed, which is used to solve the problems of artificial neural network training and feature selection (Bacanin et al., 2022). To predict the number of COVID-19 cases, a novel technique that combines machine learning and beetle antennae search methods is proposed, providing an effective technical support for controlling the outbreak of the epidemic (Zivkovic et al., 2021). A new deep neural network with transfer learning in remote object detection from drone is proposed, where the use of migration learning accelerates the training process and improves the generalization ability of the model (Woźniak et al., 2022). Although these articles may require further personalization to suit the specific research needs, they provide us with useful experiences and methods that can be fully utilized in further studies. The fusion of deep learning and image processing algorithms to detect and count banana strings method is proposed which combines the advantages of deep learning and image processing to improve the efficiency of counting (Wu et al., 2023). Automatic and intelligent data collectors and classifiers have been proposed. It is used for data collection, detection and classification of pearl millet rust and rice blast disease (Kundu et al., 2021). The articles provide a variety of techniques available for leaf and disease segmentation.

In recent years, convolutional neural networks have made significant advancements in leaf and disease detection, which is an end-to-end learning approach (Shi et al., 2023). It can automatically extract advanced image features and reduce the need for human intervention (Lu et al., 2023a).Convolutional neural networks demonstrate strong generalization capabilities, thus holding great potential for applications in disease detection (Liu et al., 2017). Full convolutional neural networks (Long et al., 2015) achieved pixel-level classification for the first time. Furthermore, the adaptability and transferability of various versions of DeepLab (Chen et al., 2017) and UNet (Ronneberger et al., 2015) have attracted a large number of researchers. A Survey of Deep Convolutional Neural Networks Applied for Prediction of Plant Leaf Diseases was raised. The article details summarize the advantages and disadvantages of different deep learning techniques for the agricultural sector (Dhaka et al., 2021). It is able to cope with the task of segmenting different diseases and is highly effective in dealing with simple environmental segmentation. In indoor environments, the segmentation of apple leaves and diseases performs well. However, in outdoor environments, the segmentation result on leaves and diseases is not satisfactory, due to the interference of light and overlapping.

With the application of relevant deep learning methods, more and more scholars have conducted extensive research on their application in apple leaf and disease segmentation (Wani et al., 2022). A fully automatic segmentation method for plant leaf images in complex environments was presented (Gao and Lin, 2019). A simple and effective semantic segmentation architecture based on a composite backbone, where OTSU was used to obtain a binary image (Yan et al., 2023b). CoAtNet integrates the attention mechanism of transformers into convolution operations for segmenting cotton leaves. Detection and classification of citrus leaf diseases based on MobileNet and self-structuring was introduced (Barman et al., 2020). The method incorporates channel attention (CA) mechanism into the ShuffleNet architecture and uses squeeze-and-excitation blocks as the CA mechanism to enhance the performance of ShuffleNet in grape leaf segmentation (Tang et al., 2020). The research of the mentioned has achieved good performance in leaf segmentation. However, in outdoor environments, diseases on the leaves cannot be accurately identified. Wang Y et al. put forward a lightweight single-stage network, which named as MGA-YOLO (Wang et al., 2022b). Based on the AlexNet model, Fu uses dilated convolution to extract coarse-grained features of diseases in the model, and extracts apple leaf diseases at multiple scales (Fu et al., 2022). A two-stage DeepLabv3+ with adaptive loss is introduced, which incorporates receptive field block and reverse attention modules, and adjusts the speed of dilated convolutions in atrous spatial pyramid pooling (ASPP) for the segmentation of apple leaf images in complex scenes (Zhu et al., 2023a).The ALDD-YOLO lightweight apple leaf disease detection model has been raised, which introduces Mobilenet-v3s to compress model size (Xu and Wang, 2023). The EADD-YOLO by improving lightweight YOLOv5 was presented. It reconstructs the backbone network with lightweight inverted residual modules and introduces them into the network to reduce feature extraction and fusion, thereby improving the efficiency of segmenting leaves (Zhu et al., 2023b). The above papers all focus on the segmentation of apple leaves and diseases from the lightweighting, and they perform well in real-time detection on mobile devices, but there may be shortcomings in disease segmentation. A lightweight dense scale network (LDSNet) for corn leaf disease classification and recognition was proposed by Zeng Y et al (Zeng et al., 2022), using different expansion rate convolutions and attention fusion methods to improve the recognition of leaves and diseases. The apple leaf and disease segmentation recognition model based on a hybrid loss function and the Convolutional Block Attention Module (CBAM) was proposed (Zhang et al., 2023). The Swin Transformer is a network model for enhancing data and identifying cucumber leaf disease (Wang et al., 2022a). An enhanced TransUNet deep learning network was posed for recognizing rice leaves (Yan et al., 2023a). An improved DeepLabv3+ deep learning network structure for segmenting grape leaf black rot has been proposed (Yuan et al., 2022). The above method is suitable for single background and high-resolution apple leaf disease images, but it does not perform well in mixed environments.

Based on the above discussion, The advances of this paper are that it proposes an improved UNet that has residual attention and an atrous spatial convolutional pooling pyramid with weight compression loss. The primary task of the proposed network is to address the pixel-scale imbalance problem that exists in mixed scenes, especially when the network captures apple leaf and disease images. Accurate segmentation of leaves and diseases provides reliable technical means for precise analysis of apple health and helps to improve the intelligence and efficiency of orchard management.

The main novelties of this work are as follows:

•To overcome the imbalance in pixel representation between leaves and diseases in mixed environments, the weighted compression loss function includes a variable modulation factor before CE, enhancing the network’s sensitivity to diseases during the training process.
•Res_CBAM is formed by integrating the residual structure with CBAM. The proposed Res_CBAM allows the network to capture multi-layered disease features and pay more attention to disease edge pixels.
•The improved ASPP structure allows the model to capture contextual information through multiscale receptive fields and different sampling rates, thereby enhancing its performance in the segmentation of diseased pixels.

The rest of the paper is structured as follows: In Section 2, the related materials and methods are presented. The material includes the obtained datasets and how to deal with them, while the methodology is a description of the details of the proposed RAAWC-UNet. The experimental results are analyzed, and the impact of network modules is discussed in Section 3. Section 4 summarizes the whole paper and makes recommendations for future research.

## 2 Materials and methods

### 2.1 Data processing

#### 2.1.1 Image datasets

Images used in the study were collected from the Northwest Agriculture and Forestry University (Northwest A&F University) Baishui Apple Experiment Station (Baishui County, Weinan City, Shaanxi Province), Luochuan Apple Experiment Station (Luochuan County, Yan’an City, Shaanxi Province), and Qingcheng Apple Experiment Station (Qingyang County, Qingyang City, Gansu Province). The apple leaf and disease datasets produced by Northwest A&F University. Most of the image were taken on a sunny day with good light, and a few pictures were collected on a rainy day. It was taken at a distance of 10-15 cm using an ABM-50OGE/BB-500GE color digital camera and an Honor V10 mobile phone. The environmental conditions include sunny, cloudy, and rainy days, and different collection environments can further enhance the diversity of the datasets.

In complex field environments, the influence of leaf characterization factors, environmental factors, and leaf disease types makes the precise delineation of leaf diseases quite difficult. (1) The influence of leaf characteristic factors: the shape, color, texture and other characteristics of leaves will affect the division of leaf diseases. For example, the disease may change the color of the leaf or cause spots, and image processing algorithms can use these features to detect and segment diseased areas. (2) Influence of environmental factors: Environmental factors such as light conditions, humidity, and temperature can also affect the classification of leaf diseases. Poor lighting conditions or shadows can affect the image quality and thus the segmentation of the disease. Changes in humidity and temperature may also cause water droplets to appear on the surface of the leaf, thus affecting disease segmentation. (3) Types of leaf diseases: Different types of leaf diseases may be affected by different characteristics and environmental factors. For example, some diseases may cause an overall discoloration of the leaf, while others may cause spots only on specific areas of the leaf.

**Figure 1:**
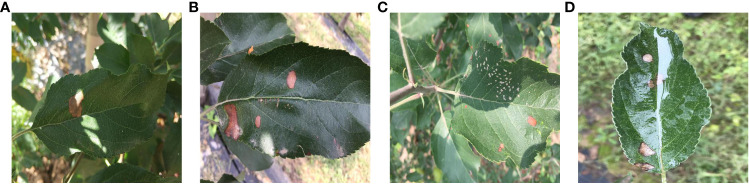
Apple leaves and diseases in outdoor environments. **(A)** Light effects. **(B)** Leaf edge folding. **(C)** wrinkled edges of the leaf. **(D)** Water droplet effects.

**Figure 1** illustrates the influence of outdoor environments on leaves. **Figure 1A** demonstrates the effect of light and shadow on apple leaves, **Figure 1B** displays folding leaves at the edges of the leaf, **Figure 1C** reveals wrinkled edges of the leaf, and **Figure 1D** shows water droplets on the leaf. Apple leaf and diseases include blotch, brown spot, grey spot, rust, and mosaic disease. The total number of original images of the four apple leaf diseases is 1866, and the resolution of the images are all 512×512 pixels. **Table 1** shows the indoor and outdoor distribution of four apple leaf disease pictures.

**Table 1:** Amount of data on apple leaf disease species.

	Alternaria blotch	Brown spot	Grey spot	Rust	Total
indoor	125	277	177	424	1003
outdoor	270	–	167	51	488
total	395	277	344	475	1491

**Figure 2:**
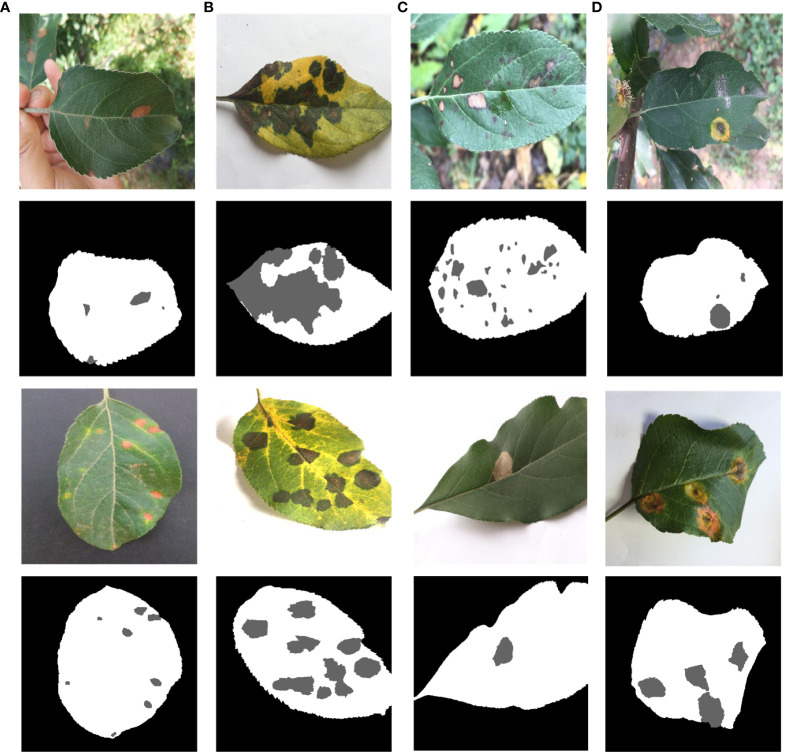
Four types of apple leaves and diseases. **(A)** Alternaria Blotch. **(B)** Brown Spot. **(C)** Grey Spot. **(D)** Rust.

**Figure 2** presents representative images of four apple leaf diseases, emphasizing the different types of diseases and distinctions. Each type of disease is selected from indoor and outdoor respectively to show the original image and label. Brown spot disease only has indoor images. The disease in **Figure 2A** is apple alternaria blotch spot, which mainly affects apple leaves, petioles, branches and fruits, producing very small brown spots on the young leaves of new shoots. The spots are often surrounded by a purple halo with clear margins. As the temperature rises, the spots can expand to 5-6 millimeters and become dark brown. The cause of alternaria blotch spot is mainly a strong virus strain of Streptomyces apples, which affects the normal growth of leaves, often resulting in twisted and wrinkled leaves, scorched parts of the disease. The disease in **Figure 2B** is brown spot, with a diameter of 3-5 millimeters. There are larger brown-green spots around the diseases, which are irregularly shaped, hence it is called green-brown disease. The pathogen of brown spot disease is caused by bivalve infestation, which leads to early defoliation of apple trees, reduces photosynthesis of apple leaves, causes malnutrition, and reduces the economic benefits of fruit growers. The disease in **Figure 2C** is a grey spot, usually 2-6 mm in diameter, with clear, reddish-brown edges that turn grey later, with small black dots scattered in the center, and is mainly caused by the pear leaf spot fungus. Leaves caused by this disease usually don’t turn yellow and fall off, but severely affected leaves will scorch, which in turn affects apple yield. The disease shown in **Figure 2D** is rust, which is initially orange-red in color and consists of small dots about 1 to 2 mm in diameter. If not controlled, the spots will grow larger and darker in the middle until they become black dots. At this time, the outermost ring of the spot is relatively light in color, and the pathogen severely damages the young fruits, resulting in the development of bad fruits.

**Figure 3:**
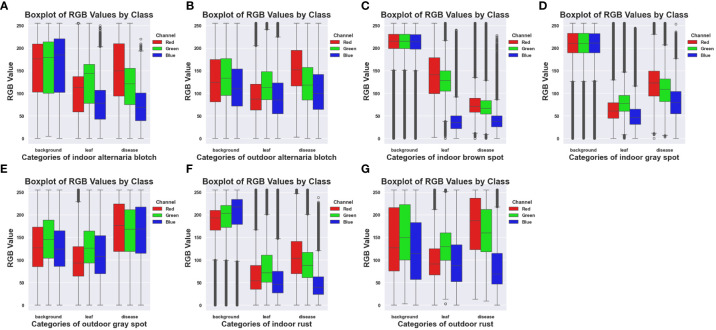
Box plots of RGB pixels of different diseases for the ALDD datasets. **(A)** Indoor Alternaria Blotch. **(B)** Outdoor Alternaria Blotch. **(C)** Indoor Brown Spot. **(D)** Indoor Grey Spot. **(E)** Outdoor Grey Spot. **(F)** Indoor Rust. **(G)** Outdoor Rust.

**Figure 3** shows the box plots of each category of the ALDD datasets, respectively analyzing the characteristics and differences of the four types of disease datasets under indoor and outdoor conditions. From **Figures 3A, B**, it can be seen that the RGB pixel values of the same type of disease are similar, the distribution range shows a decreasing trend, and the outliers of the disease are few. Due to the large difference in the pixels of the background, the RGB channel pixel values of the indoor are higher than those of the outdoor. **Figure 3C** is Brown spot, which only has indoor disease, reflecting that the pixels of the leaves and disease of brown spot are relatively complex, while the pixels of the background are relatively concentrated. **Figures 3D, E** are grey spots, and **Figures 3C, D** have the same mean value of the background pixels, and by looking at the original datasets, it can be noticed that the indoor background of the brown spot and the grey spot are close to each other. **Figures 3F, G** are Rust, the background category pixel range in **Figure 3F** is smaller than that in **Figure 3G** and relatively more concentrated.

#### 2.1.2 Image preprocessing

**Figure 4:**
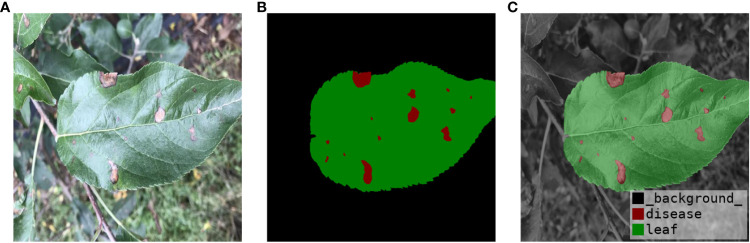
Image annotation. **(A)** Original Image. **(B)** Label Image. **(C)** Label Visualization.

**Figure 5:**
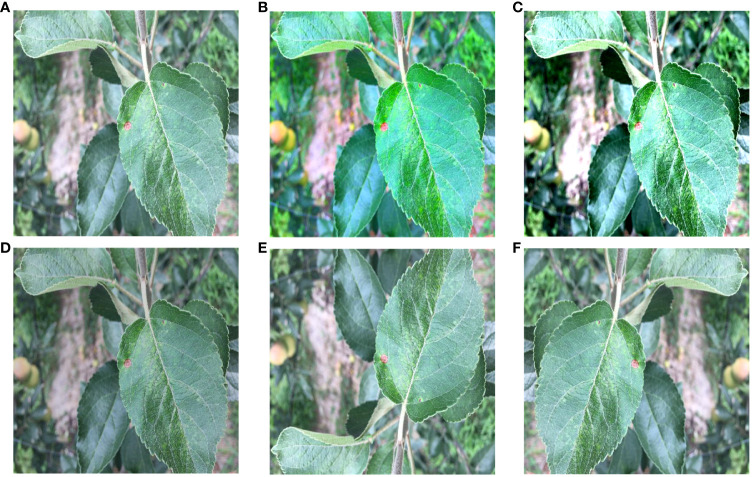
Image Enhancement. **(A)** Original image. **(B)** Color change. **(C)** Contrast change. **(D)** Brightness change. **(E)** Vertical flip. **(F)** Horizontal flip.

Firstly, preprocessing operations were performed on the images. The data on brown spots, grey spots, and rust spots in the outdoor environment were selected from the datasets. The leaves and diseases were labeled using Photoshop and Labelme, respectively, under the guidance of an apple leaf disease recognition expert. Photoshop was used to quickly mark leaves and background on apple leaves using the magic wand tool. The marking of diseases using Labelme allows for precise labeling of diseases on leaves. The final labels are saved in PNG format, which makes the labeling more precise and efficient. The labeling style is shown in **Figure 4**. Secondly, in order to avoid the overfitting problem in the later network training, improve the generalization ability of the model, and enhance diversity, the image was enhanced to simulate the complexity of the outdoor environment. Such as light intensity, light color temperature, and shadow effect. As shown in **Figure 5**, there are two main methods for image enhancement: (1) A geometric transformation of the image, which randomly flips and crops the original image horizontally and vertically. (2) Pixel transformation of the image, where the image brightness is randomly varied by 0.5–1.2 times, the contrast is randomly varied by 0.5–2.5 times, and the chromaticity is randomly varied by 0.5–2.5 times.

Then, color augmentation were applied to the indoor apple leaf datasets, increasing it from 1,003 images to 2,006 images. The outdoor datasets was expanded by randomly changing brightness, contrast, and color. The outdoor apple datasets was expanded from 488 to 1,952 sheets. This ensured that the indoor-to-outdoor ratio was as close to 1:1 as possible, thus reducing experimental error. The major apple leaf diseases included in the datasets were alternaria, brown spot, grey spot, and rust. **Figure 5** illustrates the five enhancement methods for apple brown spots as an example.

### 2.2 The proposed method

In this section, we present the proposed framework called RAAWC-UNet and the components of each module. Residual convolutional block attention module and atrous spatial pyramid pooling improved UNet with weighted compression loss is simplified as RAAWC-UNet. The proposed network mainly consists of two improved modules and the proposed loss function. Residual and CBAM are combined into Res_CBAM, and ASPP is improved into a module suitable for leaf datasets segmentation with the proposed weight compression loss.

#### 2.2.1 RAAWC-UNet framework

The outdoor image is affected by light, water droplets, bad weather, and the overlap of some outdoor leaves and target leaves, which leads to the difficulty of segmenting outdoor leaves and diseases. UNet performs well when dealing with single background leaf and disease segments. However, its performance is slightly lacking when coping with small target segments, such as diseases on apple leaves. On the one hand, the reduction of feature maps in UNet’s downsampling process can decrease the amount of information for small targets. On the other hand, during the decoding process of UNet, information is recovered from lower-level feature maps. However, this process may lose some global information, leading to the loss of context information in different regions. Therefore, to overcome this difficulty, more powerful feature extraction and detail preservation mechanisms need to be introduced into UNet to better handle these challenging small target segmentation tasks.

In order to improve the accuracy and robustness of apple leaf and disease segmentation, this study introduces an improved model on UNet, namely RAAWC-UNet. In this model, we use UNet as the backbone network and make a series of improvements based on it to better adapt to the challenging requirements of small target segmentation.

In the RAAWC-UNet model, the main improvement is in the convolutional blocks in the encoder part. The Residual and CBAM modules were introduced and integrated to form the Res_CBAM module. The Residual structure not only maintains the stable transmission of features but also helps to retain the detailed information in the network. It enables the network to be better adapted to the segmentation of small target areas, which are diseases. The CBAM makes the network more attentive to the pixel regions in the image that are decisive for classification while ignoring insignificant regions in both the channel and spatial dimensions. The merged Res_CBAM blocks help to integrate high-level and low-level semantic features, thus effectively combining detailed information with the global context. The model can better understand the features of the diseased region and improve the accuracy and robustness of segmentation.

**Figure 6:**
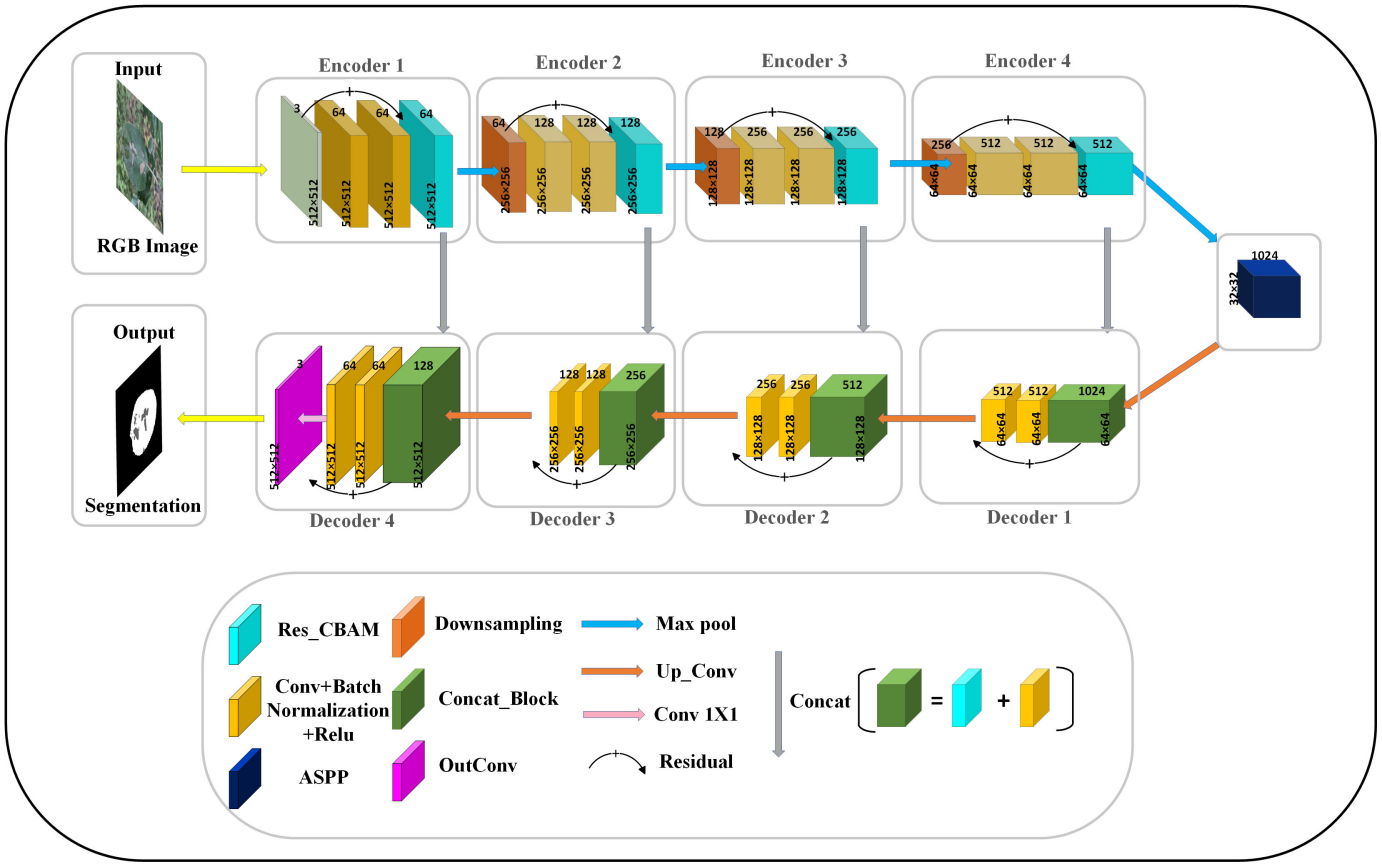
The general architecture of RAAWC-UNet with 4 encoders and 4 decoders, encoding and decoding are connected by a modified ASPP. The downsampling block, the convolutional block and Res_CBAM form an encoder, and the decoder consists of a convolutional block connected by Concat and residuals.

In addition, we replace the last downsampling layer with ASPP. The ASPP module can cover image features of different sizes within the perceptual region by utilizing different hollow rates of multiscale convolutional operations. It captures fine-grained information in leaf and disease images while also capturing a wider range of contextual relationships. Applying the modified ASPP structure to the last layer of downsampling not only improves ability to segment but also enhances generalization to a wide range of complex field scenarios. Overall, the improvement greatly enriches the model’s ability to understand the disease, thus enabling it to exhibit higher accuracy and robustness in pixel-level image segmentation. The network architecture of the proposed RAAWC-UNet is shown in **Figure 6**. The specific parameters of the proposed network structure are shown in **Table 2**. The proposed model can be reconstructed based on the structure of the proposed network diagram and the detailed operational parameters in the table.

**Table 2:** Parameters of each module in the proposed model.

Layer	Operation	Size	Output
Encoder1	Input Conv×2 Residual Res_CBAM	3×512×512 3×3, 3×3 3×3, 1×1 1×1, 7×7	- 64×512×512 64×512×512 64×512×512
Encoder2	Downsampling Conv×2 Residual Res_CBAM	2×2, stride=2 3×3, 3×3 3×3, 1×1 1×1, 7×7	64×256×256 128×256×256 128×256×256 128×256×256
Encoder3	Downsampling Conv×2 Residual Res_CBAM	2×2, stride=2 3×3, 3×3 3×3, 1×1 1×1, 7×7	128×128×128 256×128×128 256×128×128 256×128×128
Encoder4	Downsampling Conv×2 Residual Res_CBAM	2×2, stride=2 3×3, 3×3 3×3, 1×1 1×1, 7×7	256×64×64 512×64×64 512×64×64 512×64×64
ASPP	Dilation_Conv Downsampling	Rate: 6, 12, 18 2×2, stride=2	1024×64×64 1024×32×32
Decoder1	Up_Conv Concat Conv×2 Residual	Scale_factor=2 - 3×3, 3×3 3×3, 1×1	512×64×64 1024×64×64 512×64×64 512×64×64
Decoder2	Up_Conv Concat Conv×2 Residual	Scale_factor=2 - 3×3, 3×3 3×3, 1×1	256×128×128 512×128×128 256×128×128 256×128×128
Decoder3	Up_Conv Concat Conv×2 Residual	Scale_factor=2 - 3×3, 3×3 3×3, 1×1	128×256×256 256×256×256 128×256×256 128×256×256
Decoder4	Up_Conv Concat Conv×2 Residual Output	Scale_factor=2 - 3×3, 3×3 3×3, 1×1 1×1	64×512×512 128×512×512 64×512×512 64×512×512 3×512×512

#### 2.2.2 Res_CBAM module

**Figure 7:**
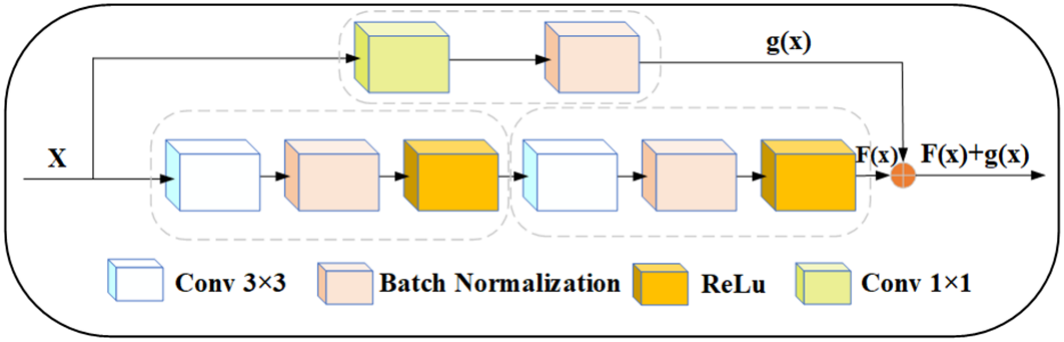
Modified residual structure, x represents the input, and x goes into two convolution blocks and a 1×1 convolution block, respectively. The output F(x) and g(x) tensors have the same dimension, and then F(x) and g(x) are summed up.

Residual module is a type of building block commonly used in deep convolutional neural networks, originally proposed by residual network in 2015 (Yang et al., 2023). As shown in **Figure 7**, the module aims to solve the problem of vanishing and exploding gradients during training, and the skipping mechanism of the residual module allows UNet to capture feature information at different levels. For apple leaf and disease segmentation, different levels of features can provide rich contextual information to accurately distinguish between diseased and normal regions. UNet can combine low-level detailed features with high-level semantic features to obtain better segmentation results. In addition, since leaf and disease segmentation tasks are very sensitive to edge information, the residual can provide better edge retention.

In this study, the UNet encoder adds the residual structure. The residual network was proposed by (He et al., 2016). To ensure that the size of the feature map obtained after two convolution operations is the same as the size of the skip connection output, we use a 1 × 1 convolution kernel to tune the channels. Thus, if the input is represented by *_x_*, the modified Res output is represented by a function: *f*(*x*) + *g*(*x*).

The following are typical representatives of channel and spatial attention:

(1) The Squeeze-and-Excitation (SE) block (Deng et al., 2023) is a network module proposed in 2018. The SE module first averages the input feature maps globally and secondly compresses the information from each channel into a scalar value. Finally, it is processed using the nonlinear activation function ReLU. The SE fundamentally removes the weights of each channel.
(2) The Efficient Channel Attention (ECA) block aims to improve the computational efficiency of channel attention (Yuan et al., 2022). The core of ECA is to adjust the importance of channel features by introducing an adaptive weight on each channel. A different convolutional kernel is applied on each channel to adaptively compute the attention weights for each channel, and these weights need to be normalized. To create a weighted feature map, the original feature map is dot-multiplied with the normalized weights. The weighting enhances the attention to the important channels and thus improves the discriminative properties of the features. The ECA is not designed for global average pooling, so it is more computationally efficient than SE.
(3) The CBAM is the attention module proposed by (Woo et al., 2018). There are two main directions for modules that improve network performance: channels and spatial attention. The channel module is similar to the channel of SE. The spatial module aims to weight different spatial locations to capture key parts of the image. All of this information is then fused through a convolutional layer to generate spatial attention weights.

**Figure 8:**
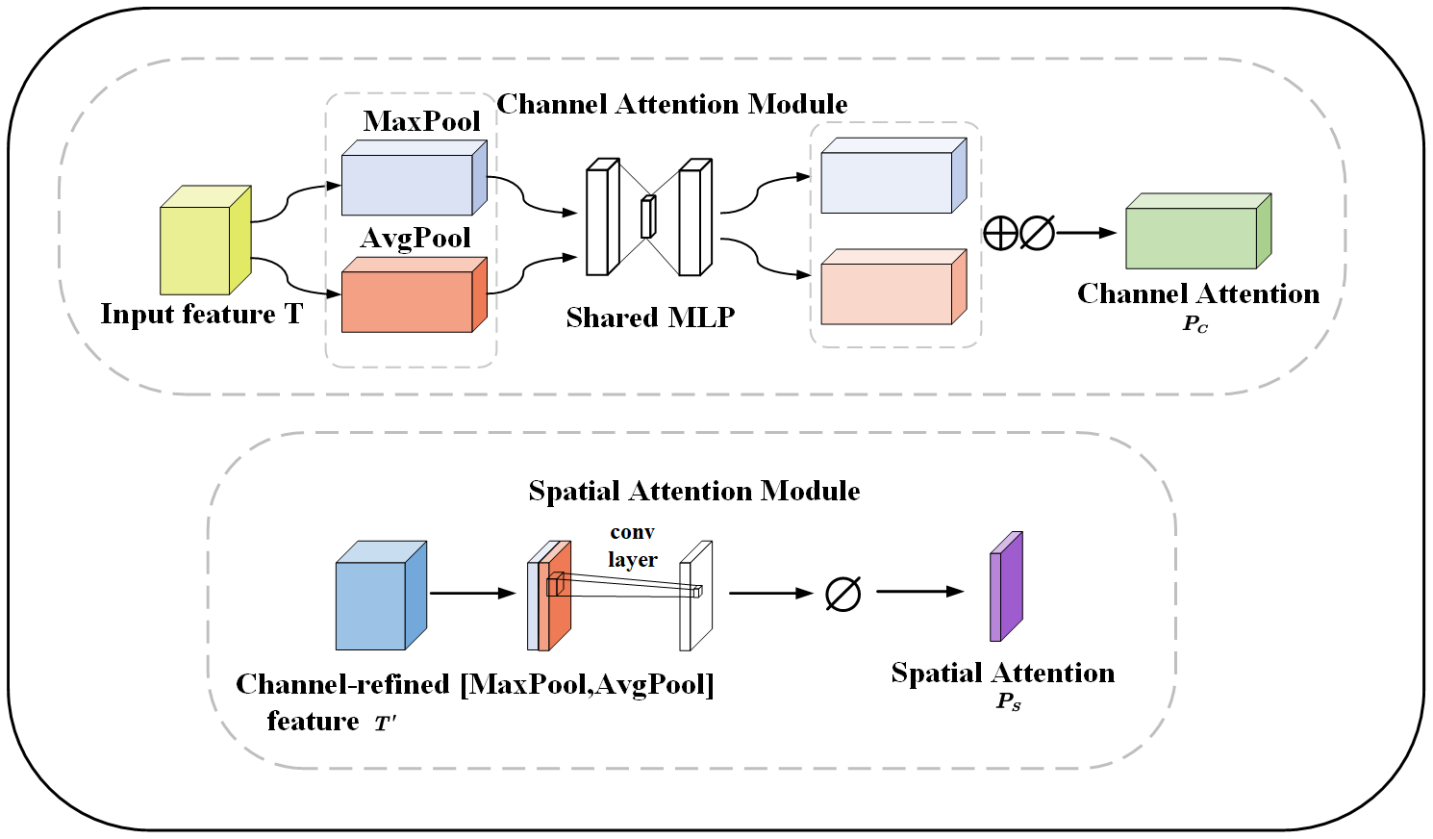
The channel and spatial attention module, MaxPool and AvgPool represent global maximum pooling and average pooling respectively. Shared MLP represents shared fully connected layers. ⊕ represents addition, ⊘ stands for sigmoid operation. [MaxPool, AvgPool] represents concatenation of the global maximum pooling and average pooling.

In order to make the network more focused on the disease and ignore irrelevant background information. The CBAM is added to the downsampling of the network. The CBAM consists of channel and spatial attention modules. The channel attention module reinforces the channel features of the disease and adaptively selects the channel features related to the disease to better capture the boundary of the disease. The spatial attention module focuses on the spatial dimension and extracts key features. Feature maps are generated through pooling operations to highlight the spatial locations of leaves and diseases. The spatial attention module helps the model better adapt to different lighting and shading situations. **Figure 8** illustrates the channel and spatial attention module. The CBAM module combines channel and spatial information to enhance the expressive and sensory capabilities of the model. It improves the performance, generalization, and interpretability of the model. This provides important applications for apple leaf and disease segmentation.

Given a feature map $T\in {\mathbb{R}}^{C\times 1\times 1}$ as input, the 1D channel feature map $P_{c}\in {\mathbb{R}}^{C\times 1\times 1}$ and the 2D spatial feature map $P_{s}\in {\mathbb{R}}^{C\times 1\times 1}$ , are derived sequentially (Equations 1–3):


(1) $$T^{\prime }=P_{c}\left(T\right)⊗T,$$

(2) $$T^{\prime \prime }=P_{s}\left(T^{\prime }\right)⊗T^{\prime },$$

(3) $$O=T+T^{\prime }+T^{\prime \prime }$$

**Figure 9:**
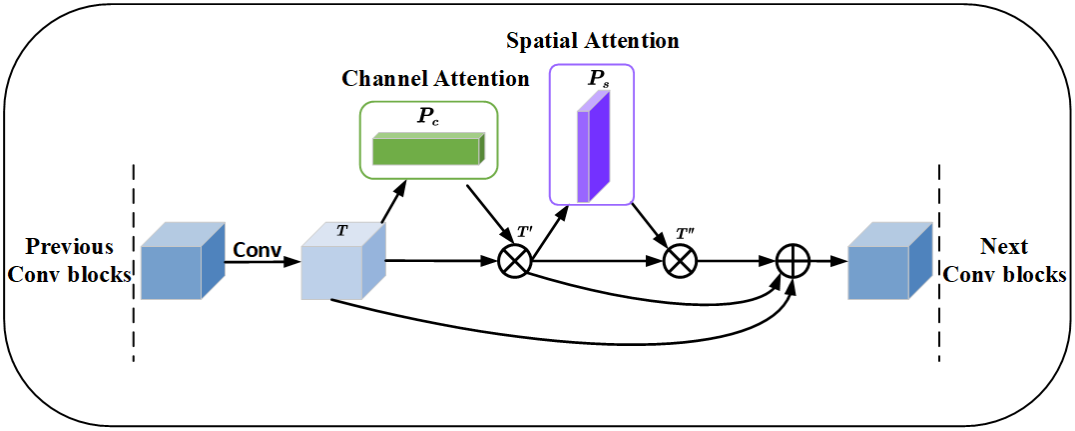
Residual convolutional block attention module, where ⊗ denotes the corresponding element multiplication and ⊕ represents the element addition, before the multiplication operation, the channel attention and spatial attention need to be broadcast according to the spatial dimension and channel dimension respectively.

⊗ represents the multiplication between elements. The essence is to perform a broadcasting mechanism. $T^{\prime \prime }$ is the output after passing through channel attention and spatial attention. *O* is the output after residual attention. The Res_CBAM involves connecting the features before attention, the features enhanced by channel attention, and the features with spatial attention on top of channel attention through residual connections. The Res_CBAM example diagram is shown in **Figure 9**.

The feature map fused different levels of feature information through the residual structure. The feature map improves the accuracy of apple leaf disease segmentation through channel attention and spatial attention, emphasizes the key features, and enhances the segmentation results.

#### 2.2.3 Modified ASPP module

**Figure 10:**
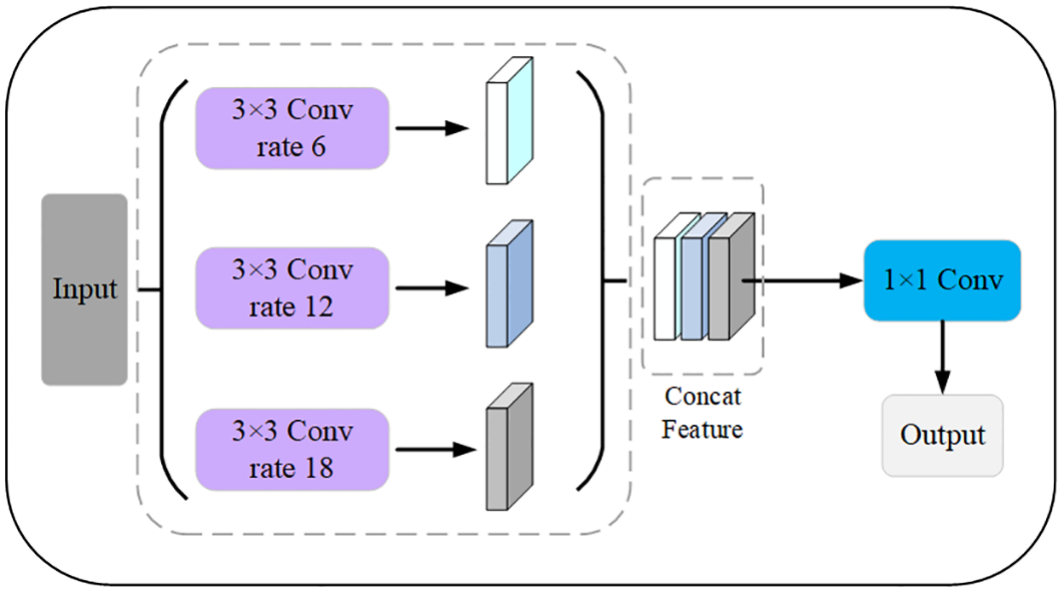
Modified ASPP module. The expansion factors of the 3 pooled pyramids are 6, 12, and 18, respectively, which are concatenated after passing through the feature layer of the pooled pyramid, and then the number of channels is varied using a 1×1 convolution.

ASPP is commonly used in image semantic segmentation tasks (Wang et al., 2021). The ASPP module is designed to help the network perceive information from different sensory fields. It operates with multiple parallel convolutional branches with different sampling rates to extend the receptive field and capture information at different scales without introducing additional parameters. The common sampling rates include 3, 6, 12, 18, etc., with larger sampling rates providing a wider range of contextual information and smaller sampling rates retaining more details. The output of the ASPP module is usually a splice or overlay of the outputs of the branches. The model can then acquire contextual information at different scales to better understand the image content and perform accurate segmentation. The modified ASPP structure removes the Conv1 × 1 and pooling operations and only keeps three Conv3 × 3 with different expansion rates. Therefore, it is possible for the modified ASPP to significantly reduce the number of parameters and the amount of float computation while still maintaining the ability to efficiently extract leaf and disease features. The modified ASPP structure is shown in **Figure 10**.

#### 2.2.4 Weight compression loss

Cross entropy (CE) is a commonly used loss function in segmentation networks to measure the difference between model predictions and true segmentation labels. However, CE loss has limitations when dealing with apple leaf and disease segmentation problems. On one hand, CE loss cannot effectively deal with the category imbalance problem, which may cause the model to be biased towards more healthy pixels. On the other hand, CE loss cannot capture spatial continuity, and diseased regions may be disconnected or blurred.

CE loss is the classical loss function in semantic segmentation (Jadon, 2020), which is defined in Equation 4:


(4) $$L_{ce}\left(y,p\right)=\{\begin{array}{cc} -log\;\left(p\right) & if\;y=1\\ -log\left(1-p\right) & if\;y=0 \end{array}$$

The *p* represents probability between the predicted value and the true value. To address the drawback of CE loss, the proposed loss function primarily deals with the issue of foreground and background pixel imbalance. We have named it WC_Loss. The Loss function is defined in Equation 5:


(5) $$WCL_{ce}\left(y,p\right)=\left\{\begin{array}{c} -{\left(1-arctan(p)\right)}^{\gamma }\text{log}(p)\;if\;y=1\\ -{\left(arctan(p)\right)}^{\gamma }\text{log}(1-p)\;if\;y=0 \end{array}\right\}$$

The *arctan*(*p*) maps the input probability values to (0*,π/*4), while [1 − *arctan*(*p*)] is mapping the probabilities to (1 − *π/*4,1). In contrast to the CE loss function, the weights in front of the CE loss are always 1 and are not elastic. [1 − arctan(*p*)]^2^ has the advantage of shortening the mapping range and adaptively adjusting the size of the front weights. The adjusted values of the weight compression loss function for mixed environments, leaf and disease pixel regions will be more continuous, helping the model to adapt more smoothly to difficult and easy samples during training.

For the apple leaf disease segmentation task, the proposed loss function WC_Loss has the following advantages: Firstly, in the apple leaf disease segmentation task, the pixel ratios of healthy and diseased leaves are unbalanced. The proposed loss function reduces the weight of easy-to-categorize samples and pays more attention to difficult-to-categorize samples, which effectively handles the imbalance between leaf and disease categories. Secondly, by introducing an adjustable hyperparameter *γ*, WC_Loss is able to focus on the difficult samples and improve the learning ability for apple leaf and disease samples. When *γ* = 0, WC_Loss becomes CE_Loss. Thirdly, the value of the modulation factor [1−*arctan*(*p*)] is determined by the probability *p*, which decreases as the probability *p* increases. When *p* increases, the pixels representing the leaf and the background are easy to classify. *γ* increases, the modulation factor [1 − *arctan*(*p*)] compresses. It is not the larger *γ* the better the accuracy of segmentation, but *γ* has to find a suitable value. Finally, the contribution to the overall loss reduces the effect of apple leaf samples and improves the accuracy of disease category classification. In conclusion, WC_Loss is a loss function that can effectively solve the problem of a serious imbalance in the proportion of leaf and disease pixels and improve the generalization ability of the model.

**Figure 11:**
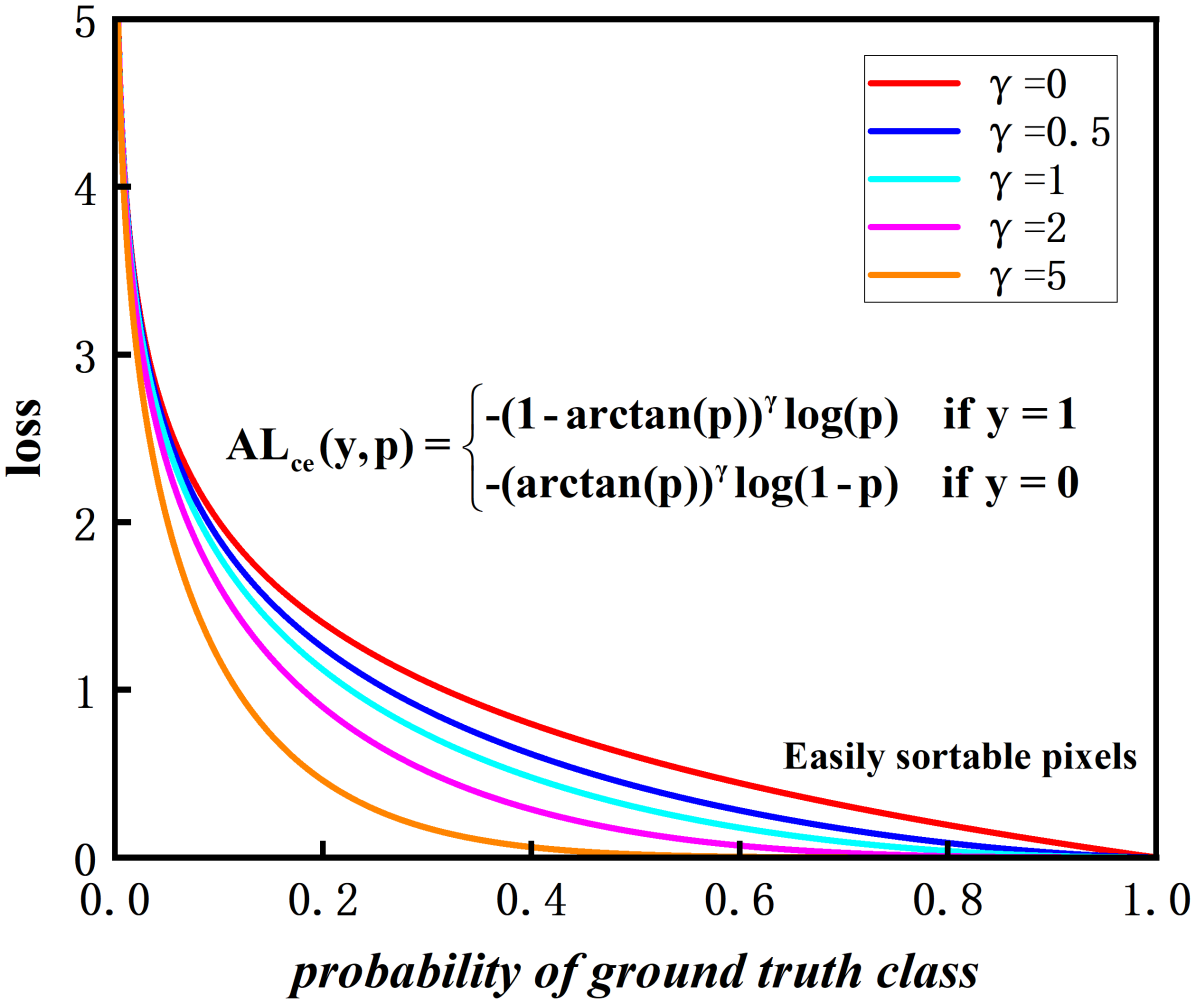
Comparison of weight compression loss and hyperparameter *γ*.

As shown in **Figure 11**, five different hyperparameters *γ* are set. As the value of *γ* increases, the possibility of fixing the probability of true labeling, the strength of the modulation factor compression increases.

The curve of the loss function shows that as the confidence level *p* increases, the loss value becomes smaller. According to the experimental results in Subsection 3.5, it is found that the best performance is achieved with *γ* = 2, combining leaf segmentation and disease segmentation.

## 3 Results and discussion

In this section, the experimental settings, evaluation metrics, and experimental results will be described in detail. The chapter includes the tuning of the loss functions of the participating designs, baseline comparisons, and ablation experiments containing individual modules. Then, the advantages and limitations of the proposed model are discussed.

### 3.1 Experimental setup

The experimental software environment is PyTorch 2.0.0 and Python 3.9. The server configuration is an Intel(R) Core (TM) i9-10900K CPU @ 3.70 GHz, 128.0 GB RAM, NVIDIA Quadro RTX 8000, CUDA 11.7. The server system is Ubuntu 18.04, and the hyperparameters are determined as follows: The initial learning rate is set to 1e-4, the number of epochs is 200, the batchsize is 4, and the model is optimized by the SGD optimizer. At the same time, the learning rate decay strategy is used to make the late gradient descent smooth and easy to converge, and the algorithm is easy to approximate the optimal solution. The experiment is done three times, and the distance of each evaluation measure is controlled at 0.0001–0.0005, which is regarded as the model being close to the optimum. The hyperparameters are set as shown in **Table 3**.

**Table 3:** Hyperparameter settings.

Hyperparameter	Values
Num_classes	3
Batch Size	4
Epochs	200
Optimizer	SGD
Learning_rate	1e-4
Momentum	0.9

In the experiments of this study, the initialization images were used with a size of 512×512×3. For effective training and evaluation. The datasets are divided into training sets, validation sets, and test sets, which are in the ratio of 6: 2: 2 and it is ensured that the datasets cover four different disease scenarios. The training sets contains 2374 images, and the validation and test sets contain 792 images each. With this division, each type of datasets contains a variety of diseases to ensure the comprehensiveness and robustness of the model.

This normalization operation not only helps to speed up the convergence of the neural network and improve generalization ability, but also effectively deals with the problem of vanishing gradients. This stage is crucial to the entire research process since it helps the model comprehend the characteristics of the images and produce predictions that are more precise.

**Figure 12:**
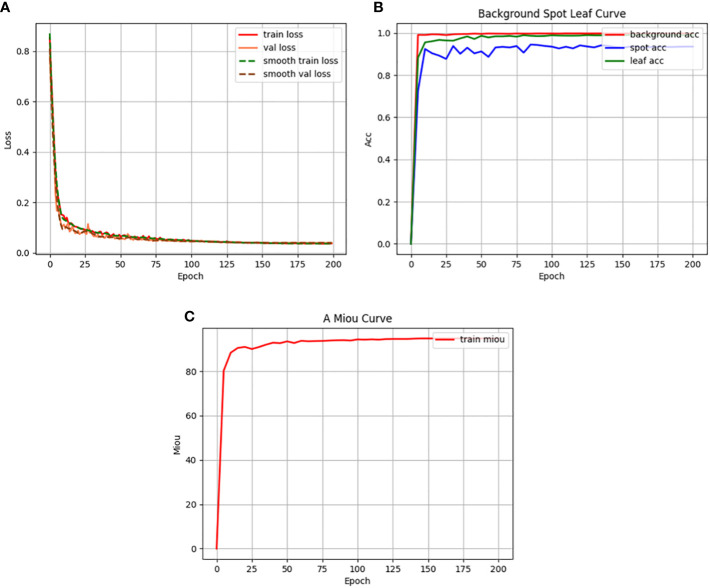
Training indicators. **(A)** Loss function curve. **(B)** Accuracy curve. **(C)** Mean intersection over union curve.

**Figure 12** illustrates the training indicators for the RAAWC-UNet model. The loss curves for the training and validation sets are shown in **Figure 12A**. **Figure 12A** shows the loss curves for the training and validation sets. It is clear from **Figure 12A** that the loss curves for both training and validation sets show a satisfactory trend. This indicates that the model performs well during the training process. The accuracy curves for the three categories of background, leaf, and disease are shown in **Figure 12B**. As seen in **Figure 12B**, the model performs admirably in terms of handling the three categories of complex background, leaves, and diseases. **Figure 12C** demonstrates the variation of mean Intersection over Union (mIoU). The model also produces good results in terms of mIoU, as can be seen in **Figure 12C**.

In summary, **Figure 12** visualizes the performance of the RAAWC-UNet model during the training process. The good performance of the loss curves, accuracy and mIoU further validates the correctness of the model for apple leaf and disease segmentation tasks.

### 3.2 Evaluation indicators

To evaluate the performance of apple leaf and disease segmentation network models in complex environments. Three types of evaluation metrics are used, which are overall performance metrics, pixel-level metrics and additional metrics for leaves and diseases.

Firstly, the overall performance evaluation metrics for the leaf and disease segmentation task were used as Accuracy (Equation 6). Accuracy is the ratio between the number of samples correctly predicted by the model and the total number of samples and is used to measure the overall predictive accuracy of the model over the entire datasets.


(6) $$Acc\;=\frac{TP+TN}{TP+FN+FP+TN}$$

Secondly, the pixel-level metrics include Precision (Equation 7), Recall (Equation 8), F1 Score (Equation 9), mPA (Equation 10) and mIoU (Equation 11). Precision is a measure of the proportion of pixels predicted to be in the positive category by the model over all the pixels labelled as positive. Recall indicates the proportion of pixels correctly marked as positive by the model over all the pixels in the actual positive category. F1 Score is a performance metric that combines Precision and Recall. It aims to balance the accuracy and the percentage coverage of the model.


(7) $$P\;=\frac{TP}{TP+FP}$$

(8) $$R\;=\frac{TP}{TP+FN}$$

(9) $$F_{1}=\frac{2*P*R}{P+R}$$

TP in the formula indicates the number of samples correctly identified as positive category, TN is the number of samples correctly identified as negative category, FP is the number of samples incorrectly identified as positive category, and FN indicates the number of samples incorrectly identified as negative category.

The mPA of accuracy category is calculated for each category at the pixel level and then averaged across all categories of accuracy. The mIoU is an important measure of the model’s segmentation performance, and its size directly reflects the model’s performance for pixel-level segmentation tasks. Where *k* denotes the total number of categories, *Pii* denotes the number of pixels predicted to be category *i* and truly belonging to category *i*, *Pij* represents the number of pixels predicted to be category *i* but really correspond to category *j*, and *Pij* indicates the number of pixels predicted to be category j but actually refer to category i. The mIoU is the pixel accuracy predicted by the model on each category and averages the results across all categories to assess the metrics. $\sum_{j=0}^{k}\;P_{ij}$ means the number of pixels of category *_i_* that are partitioned into a total of *k* categories, and $\sum_{j=0}^{k}\;P_{ji}$ means the number of pixels of category *j* that are redundantly partitioned into the i-th category.


(10) $$mPA=\frac{1}{k+1}\sum_{i=0}^{k}\frac{p_{ii}}{\sum_{j=0}^{k}P_{ji}},i=0,1,2,\ldots ,k$$

(11) $$mIoU=\frac{1}{k+1}\sum_{i=0}^{k}\frac{p_{ii}}{\sum_{j=0}^{k}P_{ij}+\sum_{j=0}^{k}P_{ji}-P_{ii}},i=0,1,2,\ldots ,k$$

Finally, the additional metric uses Cohen’s kappa coefficient (Equation 12). It is used in statistics to evaluate the consistency of a multicategorical accuracy model for background, leaf, and disease. The *po* is the empirical probability of a label assigned to any sample, and *pe* is the expected consistency between two annotators when they randomly assign labels.


(12) $$\kappa =\left(p_{o}-p_{e}\right)/\left(1-p_{e}\right)$$

### 3.3 Discussion of different attention modules

In complex outdoor environments, leaf images are easily affected by environmental factors. In order to accurately capture the texture boundary information of leaf diseases. The spatial attention module of CBAM helps to extract information at different scales in the image, better capturing the boundaries and spatial distribution of the diseases. SE and ECA segment the leaves and diseases only from the channel. CBAM has strong performance in two dimensions, it performs better in segmenting apple leaves and diseases.

The comparison results of objective indicators using different attentional mechanisms are shown in **Supplementary Table 1**. It can be seen that the addition of the SE, ECA and CBAM modules increases the overall accuracy of the network on the validation sets by 0.36%, 0.31%, and 0.39%, and the IoUs of the apples leaf pixel segmentation on the test sets by 0.83%, 0.84%, and 1.21%, and that of the disease pixel segmentation by 1.22%, 1.57%, and 3.9%. The mPA and mean precision on the test sets increased by 1.44%, 1.53%, 1.7% and 0.83%, 0.81%, 0.96%. **Supplementary Table 1** reveal that the encoder needs to consider not only channel attention but also spatial attention during feature extraction. It shows that the attention mechanism of the model plays a key role in both channel and spatial aspects in a particular task.

**Supplementary Figure 1** shows the results of image segmentation using different attention modules. By comparing **Supplementary Figures 1C–F**, it can be observed that the ability of the model to extract small lesions is enhanced with the addition of the attention module. The comparison of **Supplementary Figures 1D–F** shows that SE and ECA attention modules only have channel attention to extract a small number of minor illnesses pixels. CBAM has both channel and spatial attention and will be more comprehensive in extracting small diseases. According to the experimental results, CBAM performs better in apple leaf and disease segmentation compared to the other two attention modules. The objective results presented in **Supplementary Table 1**, as well as the multiple subjective validations in **Supplementary Figure 1**, indicate the relative superior performance of CBAM in the segmentation tasks of leaves and diseases. The effectiveness of CBAM in improving the model’s ability to focus on image details and specific regions is emphasized.

### 3.4 Discussion on hyperparametric optimizers and learning rate

Through the comparative analysis of three sets of experiments, the best choices of learning rate and optimizer were determined. The results of the experiments are shown in **Supplementary Table 2**. In the experiments, 1e-4 and 5e-4 were tried as two different learning rates, while two optimizers, SGD and Adam, were used. The experimental results show that leaf and disease segmentation perform better when the learning rate is set to 1e-4. Although the Adam optimizer can achieve faster model convergence, the stability of the results is poor, so SGD is finally chosen as the optimizer.

### 3.5 Discussion on the hyperparameter γ

To verify the effectiveness of the weight compression loss, we performed integration using Res_CBAM and a modified ASPP to validate the equation of the modulation factors. The experiment was set up for five groups and the hyperparameter *γ* was changed regularly to find the most appropriate hyperparameter configuration.

Theoretically, a larger value of *γ* indicates that the model pays more attention to the difficult-to-categorize disease pixels and ignores the samples of relatively easy-to-categorize leaves during the training process. But in practical applications, as shown in **Supplementary Table 3**, when the *γ* value is too large, it often leads to the occurrence of overfitting phenomenon, which negatively affects the model’s performance. Therefore, when weighing the importance of difficult samples and the generalization ability of the model, it is crucial to choose the *γ* value reasonably. From **Supplementary Table 3**, it can be seen that when the *γ* value is 2, the model performs optimally.

### 3.6 Ablation experiments

In this subsection, five sets of experiments are designed to validate the high accuracy of the proposed network. The control variable method is adopted to test the effectiveness of each module in the network to extract leaf and disease.

As shown in **Supplementary Table 4**, the baseline for Test 1 is UNet. For Test 2, the CE loss used in Test 1 is replaced with the proposed weight compression loss. Tests 3 and 4, based on Test 2, add the Res_CBAM module and the modified ASPP module, respectively. Test 5 integrates Res_CBAM and revised ASPP, and the loss function uses our proposed weight compression loss.

Comparing test 1 and test 2 it can be observed that the IoU of leaves and diseases increased by 0.54% and 3.96% respectively, on the test sets after using the weighted compression loss function. The overall mPA and mean precision increased by 1.51% and 0.65%. It can be seen that the imbalance in the proportion of leaf and disease pixels can be effectively dealt with after using the weighted compression loss. Comparison between Test 2 and Test 3, the IoU of leaf and disease increased by 0.84% and 0.94%, and the overall mPA and mean precision increased by 0.26% and 0.31%, which shows that Res_CBAM improves the model’s ability to express and perceive leaf and disease. Comparing Test 4 and Test 2, the last layer of the model is replaced with a modified ASPP, the IoU for leaves and diseases increased by 1.31% and 1.38%, and the overall mPA and mean precision increased by 0.38% and 0.54%. It enables the model to integrate different scales of semantic information and improves the adaptive ability of the network. Test 5 was compared with Test 4 and Test 2. After adding Res_CBAM and modified ASPP, the model’s IoU values for leaves and diseases were higher than when only Res_CBAM or ASPP were added.

**Supplementary Figure 2** show the original and ground truth images. The weight compression loss can be observed by comparing **Supplementary Figures 2C, D**, which improves the extraction of disease information. Based on the segmentation results of **Supplementary Figures 2E–G**, the following conclusion can be drawn: The model with the blend Res_CBAM and modified ASPP module performs better in leaf and disease segmentation extracted from the outdoor environment.

To better understand the performance of the proposed model, the model was validated on the test sets and the confusion matrix was plotted, which can intuitively show the prediction results of the classification model on each category. **Supplementary Figure 3** presents the confusion matrix for the three types of pixel value percentages, where it can be observed that the percentage of pixels for the disease category is lower than that of the background and leaves. In the confusion matrix, the disease category Ground Truth was incorrectly predicted as leaves with 7.72% overall. It indicates that there is still some difficulty in segmenting the small size of the disease.

**Supplementary Table 5** objectively shows the accuracy of the proposed network in each category of pixels on the four disease test sets. From **Supplementary Table 5**, it can be observed that the Brown Spot segmentation metrics are higher than Alternaria Blotch, Grey Spot, and Rust in all categories of pixels because Brown Spot exists only in indoor environments without the disturbance of outdoor environments. In contrast, Alternaria Blotch and Grey Spot segmentation metrics are relatively low, not only with the interference of background factors, but also with smaller and more diseased pixels.

### 3.7 Comparison of results for different segmentation networks

To validate the performance of the proposed network for apple leaf and disease segmentation in mixed environments, the results of the proposed network were compared with those of other different networks on the same datasets. FCN model (Gao and Lin, 2019), SegNet mode (Badrinarayanan et al., 2017), PSPNet model (Zhu et al., 2021), DeepLabv3+ model (Yuan et al., 2022), SwinUnet model (Wang et al., 2022a), UTNet model (Gao et al., 2021), DFL-UNet +CBAM model (Zhang et al., 2023), and TransUNet model (Yan et al., 2023a) are selected as the comparison methods. The above network architecture and proposed method are used to compare the effects of leaf and diseases segmentation on the same datasets. In the following experiment, we used five objective evaluation indicators, including mIoU, mPA, mPrecision, mRecall, and accuracy, to compare the performance of segmentation of each method. As can be seen from **Table 4**, the proposed network architecture has 87.15% IoU and 92.34% Recall on disease segmentation. **Table 4** demonstrate that the proposed network, RAAWC-UNet, outperforms some mainstream segmentation networks including FCN, SegNet, PSPNet, ENet, Deeplabv3+, Swin-UNet, UTNet, DFL-UNet +CBAM, and TransUNet. It increased the IoU for leaf segmentation by 2.02%, 0.69%, 0.8%, 1.48%, 0.63%, 4.29%, 3.76%, 0.21%, and 7.68%. Additionally, the IoU for disease segmentation increased by 12.18%, 5.89%, 5.15%, 8.93%, 4.9%, 8%, 4.59%, 0.83%, and 7.96%. The precision of leaves and diseases increased by 1.15%, 0.62%, 0.42%, 0.88%, 0.33%, 2.79%, 0.89%, 0.03%, 6.14% and 6.03%, 0.5%, 2.25%, 3.93%, 2.35%, 4.72%, 1.11%, 0.53%, and 3.09% respectively. The recall of leaves and diseases increased by 0.92%, 0.07%, 0.39%, 0.63%, 0.3%, 1.65%, 0.2%, 0.15%, 1.87% and 8.71%, 6.14%, 3.76%, 6.67%, 3.37%, 4.84%, 4.99%, 0.04%, and 6.28%, respectively. The RAAWC-UNet is higher than the above-mentioned other network structures.

**Table 4:** Results of different segmentation networks on the ALDD test sets.

Model	Categories	IoU/(%)	R/(%)	P/(%)	F1/(%)	κ
FCN	Background	98.98	99.45	99.52	99.48	
	Leaf	96.33	98.37	97.89	98.12	0.9410
	Disease	74.97	83.36	88.17	85.69	
SegNet	Background	99.43	99.64	99.78	99.70	
	Leaf	97.66	99.22	98.42	98.81	0.9238
	Disease	81.26	85.96	93.70	89.66	
PSPNet	Background	99.36	99.67	99.69	99.67	
	Leaf	97.55	98.90	98.62	98.75	0.9643
	Disease	82.00	88.34	91.95	90.10	
ENet	Background	99.14	99.52	99.61	99.56	
	Leaf	96.87	98.66	98.16	98.40	0.9218
	Disease	78.22	85.43	90.27	87.78	
Deeplabv3+	Background	99.44	99.70	99.74	99.71	
	Leaf	97.72	98.99	98.71	98.84	0.9635
	Disease	82.25	88.73	91.85	90.26	
Swin-UNet	Background	97.77	98.58	99.16	98.86	
	Leaf	94.06	97.64	96.25	96.94	0.9182
	Disease	79.15	87.26	89.48	88.35	
UTNet	Background	99.21	98.42	99.73	99.07	
	Leaf	94.59	99.09	98.15	98.61	0.9304
	Disease	82.56	87.11	93.09	90.00	
DFL-UNet +CBAM	Background	99.15	99.62	99.32	99.46	
	Leaf	98.14	99.14	99.01	99.07	0.9647
	Disease	86.32	92.06	93.67	92.85	
TransUNet	Background	96.07	97.01	99.00	97.99	
	Leaf	90.67	97.42	92.90	95.10	0.9020
	Disease	79.19	85.82	91.11	88.38	
RAAWC-UNet	Background	**99.59**	**99.77**	**99.82**	**99.79**	
	Leaf	**98.35**	**99.29**	**99.04**	**99.16**	**0.9788**
	Disease	**87.15**	**92.10**	**94.20**	**93.13**	

The bold values are the maximum values of the comparison algorithm in the three categories.

**Figure 13:**
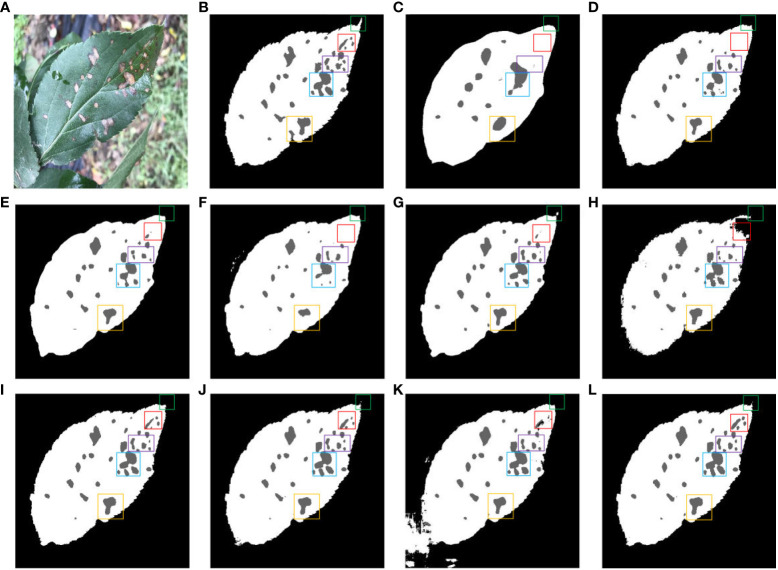
Comparison of different methods for leaf and disease segmentation. **(A)** Original images. **(B)** Ground truth. **(C)** FCN. **(D)** SegNet. **(E)** PSPNet. **(F)** ENet. **(G)** Deeplab v3+. **(H)** Swin-UNet. **(I)** UTNet. **(J)** DFL-UNet +CBAM. **(K)** TransUNet. **(L)** Proposed method.

**Figure 13** shows the impact of different segmentation models on the segmentation of dense leaves and diseases in outdoor environments. **Figure 13A** is a typical representation of leaf and dense disease segmentation under complex backgrounds. **Figure 13B** shows leaf and disease segmentation ground truth. As shown in **Figure 13C**, FCN has the worst segmentation effect, only large diseases can be segmented, and the edges of the leaves cannot be segmented with the effect of the saw tooth of the original leaves. Compared to **Figures 13D, E, G, B**, SegNet, PSPNet, and Deeplab v3+ are unable to segment small diseases in dense areas. The segmentation results of **Figures 13F, K** indicate that Enet and TransUNet incorrectly segmented overlapping leaves in an outdoor environment. As displayed in **Figures 13H–J**, Swin-UNet, UTNet, and DFL-UNet +CBAM cannot effectively segment wrinkles, wavy leaves, and diseases in dense areas. From **Figure 13L**, it can be concluded that the proposed method is better than other models for both leaf and disease segmentation in outdoor environments.

**Figure 14:**
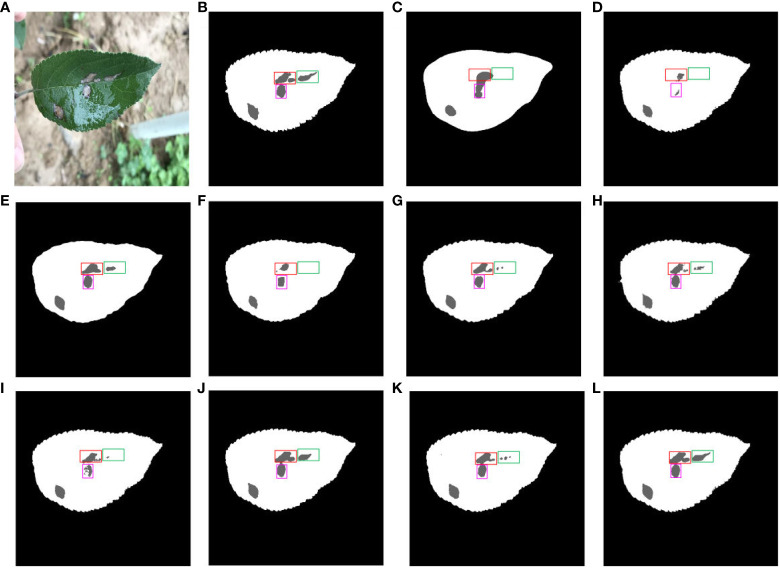
Comparison of multiple segmentation networks for leaves and diseases affected by water droplets. **(A)** Original images. **(B)** Ground truth. **(C)** FCN. **(D)** SegNet. **(E)** PSPNet. **(F)** ENet. **(G)** Deeplab v3+. **(H)** Swin-UNet. **(I)** UTNet. **(J)** DFL-UNet +CBAM. **(K)** TransUNet. **(L)** Proposed method.

**Figure 14** shows a comparison of multiple segmentation networks for leaves and diseases affected by water droplets. **Figure 14A** is a typical representation of leaf and disease segmentation under the influence of water droplets. **Figure 14B** shows ground truth for leaf and disease segmentation. Comparison of **Figures 14C, D, F, J** shows the poor effectiveness of FCN, SegNet, ENet and DFL-UNet +CBAM segmentation of long strips of disease. **Figures 14H, I** compared to **Figure 14B** show that SwinUNet and UTNet have jagged tooth-like segmentation of disease pixels. The comparison of disease segmentation between **Figures 14E, G, K** with **Figure 14B** reveals that PSPNet, Deeplab v3+, and TransUNet are not precise in segmenting water droplet-affected diseases. From **Figure 14L**, it is clear that the proposed method performs better than the other models in disease segmentation for long strips and affected by water droplets.

**Figure 15:**
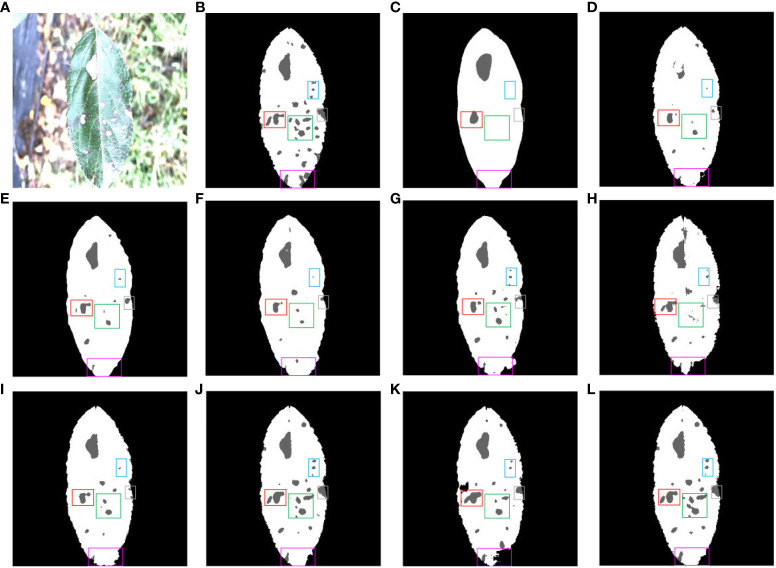
Comparison of multiple segmentation networks for leaves and diseases affected by light. **(A)** Original images. **(B)** Ground truth. **(C)** FCN. **(D)** SegNet. **(E)** PSPNet. **(F)** ENet. **(G)** Deeplab v3+. **(H)** Swin-UNet. **(I)** UTNet. **(J)** DFL-UNet +CBAM. **(K)** TransUNet. **(L)** Proposed method.

**Figure 15** shows the different segmentation models for leaf and disease segmentation affected by light. **Figure 15A** presents a typical schematic of leaf and disease segmentation under the influence of light. **Figure 15B** demonstrates the ground truth for leaf and disease segmentation. **Figure 15C** FCN has the worst results, the leaves do not have the megadentate shape of the ground truth, and **Figures 15D, H** illustrate the poor segmentation of large disease pixels in SegNet and SwinUNet under the influence of light. **Figures 15E, F, I, J** exhibit the inability of PSPNet, ENet, UTNet and DFL-UNet +CBAM to accurately segment small diseases in dense areas affected by light. **Figures 15G, K** show that Deeplab v3+ and TransUNet incorrectly segmented the leaf pixels, and **Figure 15L** displays that the proposed network segmented the leaf pixels relatively well compared to the other networks under the influence of light.

The objective results of different segmentation networks on the ALDD test sets show that the proposed network has better segmentation ability for leaves and diseases than other models in various complex environments.

### 3.8 Evaluation metrics for different segmentation networks within multiple scenarios

To further validate the performance of the proposed model and other comparative algorithms for segmentation of background, leaf and disease in different scenarios, the experiments are conducted with FCN, SegNet, PSPNet, ENet, Deeplabv3+, Swin-UNet, UTNet, DFL-UNet+CBAM, TransUNet, and the proposed RAAWC-UNet on both indoor and outdoor scenarios with Alternaria blotch, Brown spot, Grey spot and Rust.

**Supplementary Table 6** objectively shows the mIoU and mPA of the above 10 segmentation algorithms on the test sets with 7 scenarios. As a whole, the two objective indicators of our proposed model surpass those of other models, which demonstrates the superiority of our model. In addition, it is worth noting that due to the dense and widespread occurrence of Grey spot, the segmentation performance of the proposed RAAWC-UNet is comparatively the poorest among the various types of diseases. However, it is still higher than that of the other algorithms mentioned above in Grey spot segmentation.

## 4 Conclusions

In this study, the proposed RAAWC-UNet was developed for segmenting apple and leaf diseases in mixed environments by incorporating ASPP, fusing residual and CBAM modules into UNet with weight compression loss. RAAWC-UNet performs outstandingly in disease segmentation compared to other leaf and disease segmentation networks. The Res_CBAM module effectively captures features at different levels while integrating channel and spatial information. It not only enriches contextual information but also enhances the model’s perceptual capabilities, addressing the issue of foreground and background pixel imbalance. The ASPP module adapts by utilizing different dilation rates, flexibly adjusting the convolution kernel’s perceptual field to accommodate various leaf and disease pixel sizes. The weight compression loss helps with fast convergence early in the model training. The proposed method is better than most of the segmentation algorithms, and the model presents superior performance especially when dealing with small size and diverse disease segmentation tasks.

Compared with some commonly used networks, the model has lower computational complexity and fewer parameters. While, its computational complexity and parameter quantity are slightly higher in comparison of some lightweight networks. However, the model was better at the task of segmenting leaves and diseases when dealing with factors such as light and water droplets in outdoor environments.

In addition, the following research direction will focus on expanding the scope of data collection and considering various harsh environmental conditions, such as fog, rain and frost, to further study the impact of these environments on the performance of the model. Eventually, we are committed to research on model optimization to meet the needs of resource constrained environments. This effort will further promote the research and development of smart agriculture.

## Data availability statement

The datasets presented in this study can be found in online repositories. The names of the repository/repositories and accession number(s) can be found below: https://drive.google.com/file/d/1qV3zZCNh8FhrMwQwZds9rRkm9SUQXV5P/view?usp=sharing.

## Author contributions

JW: Conceptualization, Funding acquisition, Validation, Writing – review & editing. JJ: Conceptualization, Methodology, Software, Validation, Writing – original draft, Writing – review & editing. YZ: Data curation, Supervision, Writing – review & editing. HW: Visualization, Writing – review & editing. SZ: Project administration, Supervision, Writing – review & editing.
