# Supplementary material for: RAAWC-UNet: an apple leaf and disease segmentation method based on residual attention and atrous spatial pyramid pooling improved UNet with weight compression loss
Source: Front Plant Sci. 2024 Mar 11;15:1305358. doi: 10.3389/fpls.2024.1305358 (PMC10961398; doi:10.3389/fpls.2024.1305358)
Supplement: Supplementary file 1 [file DataSheet_1.pdf]

Supplementary Material

Table S1. Comparative experiments using different attentional mechanisms.

Methods	IoU/(%)			mPA/(%)	Acc/(%)	mP/(%)
	Background	Leaf	Disease			
UNet	98.67	96.44	81.54	95.15	98.86	96.31
SE-UNet	99.09	97.27	82.76	96.59	99.22	97.14
ECA-UNet	99.15	97.28	83.11	96.68	99.17	97.12
CBAM-UNet	**99.31**	**97.65**	**85.44**	**96.85**	**99.25**	**97.27**

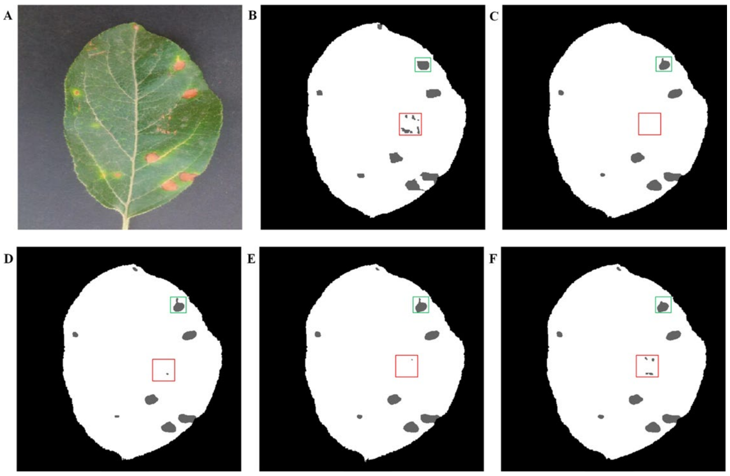

Figure S1. Comparison of image segmentation by different attention modules. **(A)** Original image. **(B)** Ground truth. **(C)** UNet segmentation results. **(D)** UNet+SE segmentation results. **(E)** UNet+ECA segmentation results. **(F)** UNet+CBAM segmentation results.

Table S2. Objective results for learning rate and optimizer type selection on the ALDD test sets.

Num_classes	3	3	3
Input_shape	[512,512]	[512,512]	[512,512]
Batch_size	4	4	4
Init_lr	1e-2	5e-2	1e-4
Optimizer_type	SGD	SGD	Adam
Momentum	0.9	0.9	0.9
mIoU	**95.03**	94.72	94.66
Acc	**99.47**	99.21	99.36

Table S3. Objective results of hyperparameters γ for different loss functions on the ALDD test sets.

γ	IoU/(%)			PA/(%)		
	Background	Leaf	Disease	Background	Leaf	Disease
0	99.03	96.98	85.90	99.44	99.19	91.94
1	99.51	98.34	86.54	99.52	99.25	92.05
2	**99.59**	**98.35**	**87.15**	**99.77**	**99.29**	**92.10**
3	99.54	98.26	86.96	99.46	99.27	92.01
5	99.55	98.20	86.87	99.37	99.24	91.94

Table S4. Ablation experiments performed on the ALDD test sets.

Test No.	Model	IoU/(%)			mPA/(%)	Acc/(%)	mP/(%)
		Background	Leaf	Disease			
1	UNet+CE loss	98.67	96.44	81.54	95.12	98.86	96.31
2	UNet+adaptive loss	99.03	96.98	85.50	96.63	97.08	96.96
3	UNet+Res_CBAM+adaptive loss	99.31	97.82	86.44	96.89	97.27	97.27
4	UNet+ASPP+adaptive loss	99.57	98.29	86.88	97.01	99.36	97.50
5	Unet+Res_CBAM+ASPP+adaptive loss	**99.59**	**98.35**	**87.15**	**97.05**	**99.47**	**97.69**

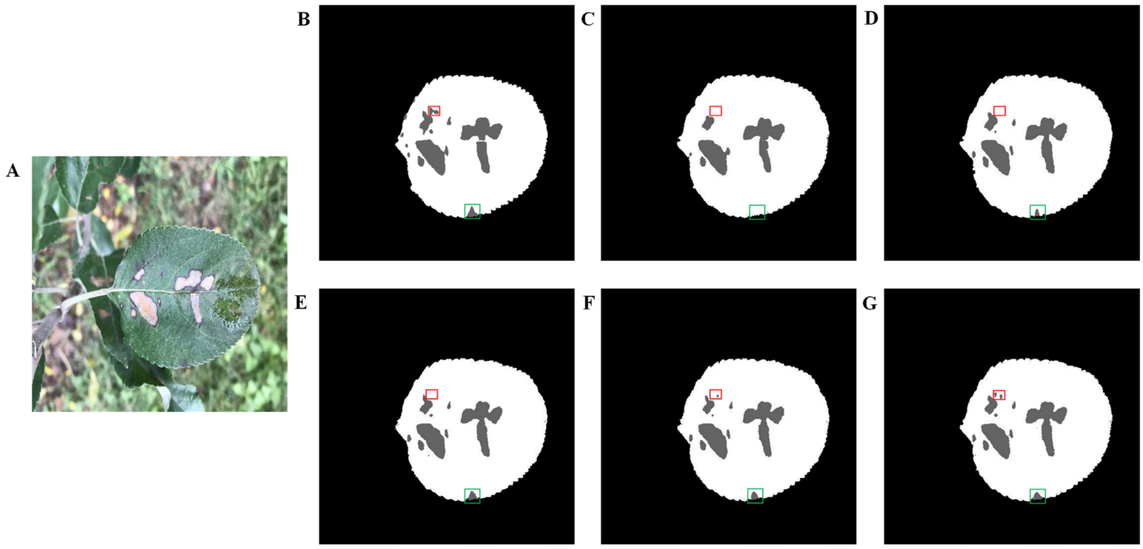

Figure S2. Results of ablation experiments. **(A)** Original images. **(B)** Ground truth. **(C)** UNet+CE. **(D)** UNet+adaptive loss. **(E)** UNet+Res_CBAM+adaptive loss. **(F)** UNet+ASPP+adaptive loss. **(G)** UNet+Res_CBAM+ASPP+adaptive loss.

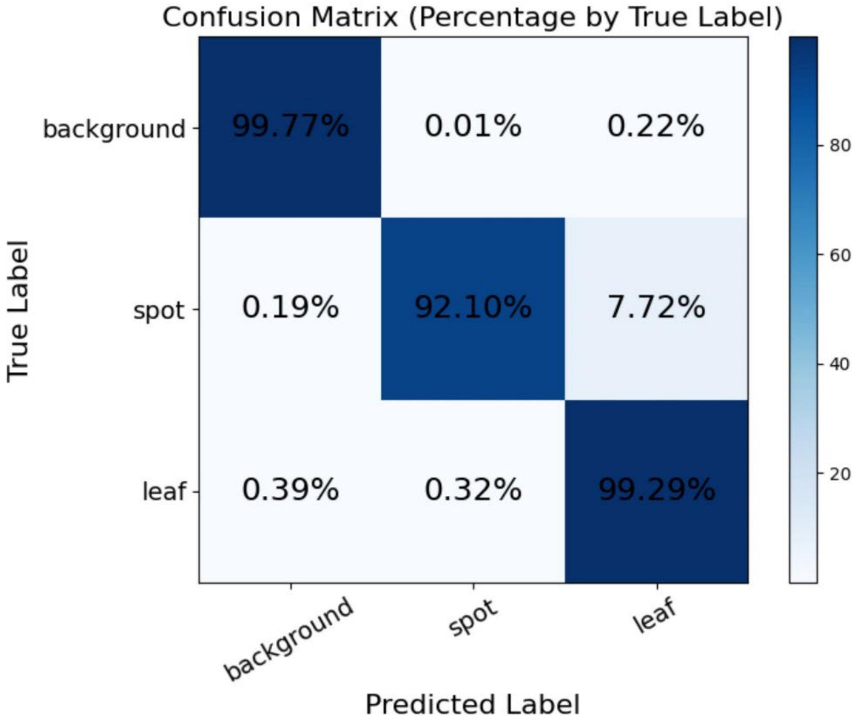

Figure S3. The confusion matrix of the proposed model.

Table S5. Objective indicators for the evaluation of different disease categories.

Disease Name	Categories	IoU/(%)	R/(%)	P/(%)	F1/(%)
Alternaria blotch	Background	99.5	99.72	99.78	99.74
	Leaf	98.70	99.43	99.26	99.34
	Disease	84.43	90.20	92.85	91.50
Brown spot	Background	99.72	99.88	99.84	99.85
	Leaf	96.63	98.35	98.22	98.28
	Disease	89.71	94.03	95.13	94.57
Gray spot	Background	98.62	99.75	99.85	99.79
	Leaf	99.12	99.49	99.12	99.30
	Disease	84.54	89.53	93.61	91.52
Rust	Background	99.64	99.80	99.84	99.81
	Leaf	98.32	99.29	99.02	99.15
	Disease	85.68	91.00	93.60	92.28

Table S6. Evaluation metrics for different segmentation networks in multiple scenarios.

Model	Indicators	Evaluation metrics for multiple scenarios/(%)						
		A_in	A_out	B_in	G_in	G_out	R_in	R_out
FCN	mIoU	87.26	88.88	91.67	87.62	87.28	93.91	94.07
	mPA	92.52	93.75	97.94	92.53	91.75	93.66	93.91
SegNet	mIoU	91.24	92.63	95.51	90.69	89.15	94.65	95.59
	mPA	93.99	96.71	97.12	92.23	90.46	97.05	96.99
PSPNet	mIoU	92.13	93.04	94.15	91.83	89.59	94.13	95.91
	mPA	95.22	95.93	97.59	94.35	91.56	96.95	97.76
ENet	mIoU	91.28	91.55	93.67	88.63	85.79	94.27	94.73
	mPA	95.74	94.9	97.77	90.98	89.78	95.63	96.93
Deeplabv3+	mIoU	92.11	93.0	94.52	91.82	89.89	94.05	96.57
	mPA	94.43	96.55	97.90	94.33	92.62	96.79	98.03
Swin-UNet	mIoU	91.7	89.2	94.12	89.09	82.35	94.54	91.2
	mPA	95.6	94.04	97.33	93.08	89.31	96.7	95.42
UTNet	mIoU	90.33	92.68	94.16	90.65	88.72	93.35	94.94
	mPA	93.27	95.93	97.51	93.02	90.82	96.56	96.99
DFL-UNet +CBAM	mIoU	94.11	94.52	95.56	91.7	94.31	94.78	96.72
	mPA	96.89	97.06	97.71	95.75	96.02	97.09	98.11
TransUNet	mIoU	91.17	86.32	94.27	87.99	82.86	93.2	84.61
	mPA	94.38	93.4	96.84	92.38	89.29	95.67	91.96
RAAWC-UNet	mIoU	**94.26**	**95.07**	**95.73**	**93.92**	**94.51**	**94.96**	**96.74**
	mPA	**96.91**	**97.17**	**97.97**	**95.89**	**96.06**	**97.15**	**98.18**
